# Supplementary material for: Large-scale seroepidemiology uncovers nephro-urological pathologies in people with tau autoimmunity
Source: PLoS Biol. 2025 Nov 26;23(11):e3003488. doi: 10.1371/journal.pbio.3003488 (PMC12685212; doi:10.1371/journal.pbio.3003488)
Supplement: S1 Table — (DOCX) [file pbio.3003488.s001.docx]

| **S1 Table. Targeted AD screen samples.** | | | |
| --- | --- | --- | --- |
|  | AD | Control | P |
| Women, n | 30 | 39 |  |
| Men, n | 17 | 29 |  |
| Age, median (IQR range) | 78 (70,5-86) | 81 (71-85) | 0.594 |
