# Supplementary material for: Large-scale seroepidemiology uncovers nephro-urological pathologies in people with tau autoimmunity
Source: PLoS Biol. 2025 Nov 26;23(11):e3003488. doi: 10.1371/journal.pbio.3003488 (PMC12685212; doi:10.1371/journal.pbio.3003488)
Supplement: S2 Table — (DOCX) [file pbio.3003488.s002.docx]

**S2 Table. Laboratory parameters used in the statistical analysis.**

| **Laboratory Parameter** | **Unit** |
| --- | --- |
| Sodium | mmol/L |
| Cholesterol, total | mmol/L |
| Chloride | mmol/L |
| C-reactive protein | mg/L |
| Iron | µmol/L |
| Cholesterol-LDL | mmol/L |
| Phosphate | mmol/L |
| Uric acid | µmol/L |
| Ferritin | µg/L |
| Glucose | mmol/L |
| Transferrin | µmol/L |
| Lipase | U/L |
| Myoglobin | µg/L |
| N-terminal pro-B-type natriuretic peptide (NT-pro-BNP) | ng/L |
| Parathyroid hormone | ng/L |
| Protein, urine | g/L |
| Protein-to-creatinine ratio, urine | g/mmol |
| Activated partial thromboplastin time (aPTT) | seconds |
| Erythrocytes | T/L |
| Mean corpuscular volume | fL |
| Mean corpuscular hemoglobin | pg |
| Reticulocytes | G/L |
| Hyperchromic erythrocytes | % |
| Hypochromic erythrocytes | % |
| Neutrophils | G/L |
| Monocytes | G/L |
| Eosinophils | G/L |
| Basophils | G/L |
| Lymphocytes | G/L |
| Large unstained cells | G/L |
| Leukocytes | G/L |
| Neutrophils | % |
| Monocytes | % |
| Eosinophils | % |
| Basophils | % |
| Lymphocytes | % |
| Large unstained cells | % |
| International normalized ratio (INR) |  |
| Red cell distribution width (RDW) | % |
| Mean platelet volume | fL |
| Fibrinogen | g/L |
| Anti-factor Xa activity | IU/mL |
| IgG | g/L |
| IgA | g/L |
| IgM | g/L |
| Cholesterol-HDL | mmol/L |
| Lactate | mmol/L |
| Albumin-to-creatinine ratio, urine | mg/mmol |
| Potassium | mmol/L |
| Amylase | U/L |
| Lactate dehydrogenase | U/L |
| Albumin | g/L |
| Aspartate aminotransferase | U/L |
| Calcium, total | mmol/L |
| Urea | mmol/L |
| Magnesium | mmol/L |
| Protein | g/L |
| Creatinine | µmol/L |
| Free triiodothyronine  (FT3) | pmol/L |
| Thyroid-stimulating hormone | mU/L |
| Glucose (fasting) | mmol/L |
| Anti-HBs | IE/L |
| Anti-HBc-IgG/IgM ratio |  |
| Anti-HCV-IgG |  |
| HIV Ag/Ab ratio |  |
| Procalcitonin | µg/L |
| Bilirubin, total | µmol/L |
| Calcium, albumin corrected | mmol/L |
| HBs-Antigen |  |
| pH, urine |  |
| Density, urine | g/mL |
| Leukocytes, urine | /µL |
| Erythrocytes, urine | /µL |
| Renal tubular cells, urine | /µL |
| Squamous epithelial cells, urine | /µL |
| Hyaline casts, urine | /µL |
| Yeast, urine | /µL |
| Thrombin time | seconds |
| eGFR CKD-EPI 2009 | mL/min/1.73m^2^ |
| Hemoglobin | g/L |
| Hematocrit | L/L |
| Mean corpuscular hemoglobin concentration | g/L |
| Hemoglobin distribution width (HDW) | g/L |
| Retikulozyten (automatisch) % | % |
| Immature granulocytes, absolute | G/L |
| Immature Granulocytes % (automatisch) | % |
| Nucleated red blood cells, absolute | G/L |
| Nucleated red blood cells | /100 Leukocytes |
| Transferrin saturation | % |
| Hemoglobin A1c, NGSP | % |
| Hemoglobin A1c, IFCC | mmol/mol |
| Gamma-glutamyl transferase | U/L |
| Alanine aminotransferase | U/L |
| Creatine kinase, total | U/L |
| Troponin T, high sensitivity | ng/L |
| eGFR BIS1 | mL/min/1.73m^2^ |
| Prothrombin time | seconds |
| non-HDL-cholesterol | mmol/L |
| Triglycerides | mmol/L |
| Alkaline phosphatase | U/L |
| Free thyroxine (FT4) | pmol/L |
| 25-hydroxyvitamin D | µg/L |
| Osmolality | mmol/Kg |
| Vitamin B12 | ng/L |
| Ferritin (at risk indivuduals) | µg/L |
| Transferrin saturation (at risk individuals) | % |
