# Supplementary material for: Large-scale seroepidemiology uncovers nephro-urological pathologies in people with tau autoimmunity
Source: PLoS Biol. 2025 Nov 26;23(11):e3003488. doi: 10.1371/journal.pbio.3003488 (PMC12685212; doi:10.1371/journal.pbio.3003488)
Supplement: S3 Table — (DOCX) [file pbio.3003488.s003.docx]

| **S3 Table. ICD-10 codes used for the grouping of neurological disorders in the statistical analysis.** | | | | |
| --- | --- | --- | --- | --- |
| **Group of Disorders** | **ICD-10 codes** |  | |  |
| Alzheimer's disease | G30, F00 |  | |  |
| Non-Alzheimer's dementia | F01, F02, F03, G31.0, G31.3, G31.82 | | | |
| Epilepsy | G40, G41 |  | |  |
| Migraine | G43 |  | |  |
| Other headache disorders | G44, G50 |  | |  |
| Meningitis, Encephalitis or Myelitis | G00, G01, G02, G03, G04, G05 | | | |
| Parkinson's disease | G20 |  | |  |
| Secondary parkinsonism | G21 |  | |  |
| Atypical parkinsonism | G23.1, G23.2, G23.3, G23.8 | | | |
| Essential tremor | G25.0 |  | |  |
| Dystonia | G24 |  | |  |
| Myoclonus | G25.3 |  | |  |
| Multiple sclerosis | G35 |  | |  |
| Neuropathies | G51, G52, G53, G56, G57, G60, G61, G62 | | | |
| Myoneural junction disorders | G70 |  | |  |
| Myopathies | G71, G72 | |  |  |
| Hereditary ataxia/spastic paraplegia | G11 |  | |  |
| Motor neuron disease | G12.2 |  | |  |
| Huntington's disease | G10 |  | |  |
| Sleep disorders | G47 |  | |  |
| Stroke | G45 |  | |  |
| Infarction | I63, G46, I69.3 | | |  |
| Hemorrhage | I60, I61, I62.9, I69.0, I69.2 | | | |
| Alzheimer’s disease | G30.0, G30.1, G30.8, G30.9, F00.0, F00.1, F00.2, F00.9 | | | |
| Frontotemporal dementia | G31.0 | | | |
| Progressive supranuclear palsy | G23.1 | | | |
