# Supplementary material for: Large-scale seroepidemiology uncovers nephro-urological pathologies in people with tau autoimmunity
Source: PLoS Biol. 2025 Nov 26;23(11):e3003488. doi: 10.1371/journal.pbio.3003488 (PMC12685212; doi:10.1371/journal.pbio.3003488)
Supplement: S4 Table — (DOCX) [file pbio.3003488.s004.docx]

| **S4 Table. CD-10 codes used for the grouping of systemic disorders in the statistical analysis**. | | | | | | | |
| --- | --- | --- | --- | --- | --- | --- | --- |
| **Group of Disorders** | **ICD-10 codes** | |  |  |  |  |  |
| Cardiovascular | Ixx |  | |  |  |  |  |
| Heart | I0x, I20, I21, I22, I23, I24, I25, I3x, I4x, I5x | | | | |  |  |
| Hypertension | I1x |  | |  |  |  |  |
| Vascular | I7x |  | |  |  |  |  |
| Digestive | Kxx |  | |  |  |  |  |
| Stomach and intestine | K2x, K3x, K4x, K5x, K60, K61, K62, K63, K64 | | | | | |  |
| Liver | K7x |  | |  |  |  |  |
| Endocrine, Metabolic & Nutritional | Exx |  | |  |  |  |  |
| Diabetes | E10, E11, E12, E13, E14 | | | |  |  |  |
| Nutrition | E0x |  | |  |  |  |  |
| Thyroid | E4x, E5x, E60, E61, E63, E64, E65, E66, E67, E68 | | | | | |  |
| Hematopoietic | D5x, D6x, D7x, D8x | | |  |  |  |  |
| Anemia | D5x |  | |  |  |  |  |
| Coagulation | D65, D66, D67, D68, D69 | | | |  |  |  |
| Infections | Axx, Bxx |  | |  |  |  |  |
| Kidney & Urinary | Nxx |  | |  |  |  |  |
| Kidney | N0x, N1x, N2x | | |  |  |  |  |
| Urinary | N3x |  | |  |  |  |  |
| Musculoskeletal | Mxx |  | |  |  |  |  |
| Neoplasms | Cxx, D0x, D1x, D2x, D3x, D4x | | | |  |  |  |
| Hematopoietic | C8x, C90, C91, C92, C93, C94, C95, C96 | | | | |  |  |
| Malignant solid | C0x, C1x, C2x, C3x, C4x, C5x, C6x, C71, C72, C73, C74, C75 | | | | | | |
| Neurologic | F0x, Gxx |  | |  |  |  |  |
| Pregnancy& Congenital | Oxx, Pxx, Qxx | | |  |  |  |  |
| Psychiatric | Fxx, Gxx |  | |  |  |  |  |
| Respiratory | Jxx |  | |  |  |  |  |
| Skin | Lxx |  | |  |  |  |  |
