# Supplementary material for: Large-scale seroepidemiology uncovers nephro-urological pathologies in people with tau autoimmunity
Source: PLoS Biol. 2025 Nov 26;23(11):e3003488. doi: 10.1371/journal.pbio.3003488 (PMC12685212; doi:10.1371/journal.pbio.3003488)
Supplement: S1 Raw Images — (PDF) [file pbio.3003488.s006.pdf]

Fig. 2F: Raw WB images with merged molecular weight markers acquired by the Vilber Fusion Solo imaging system.

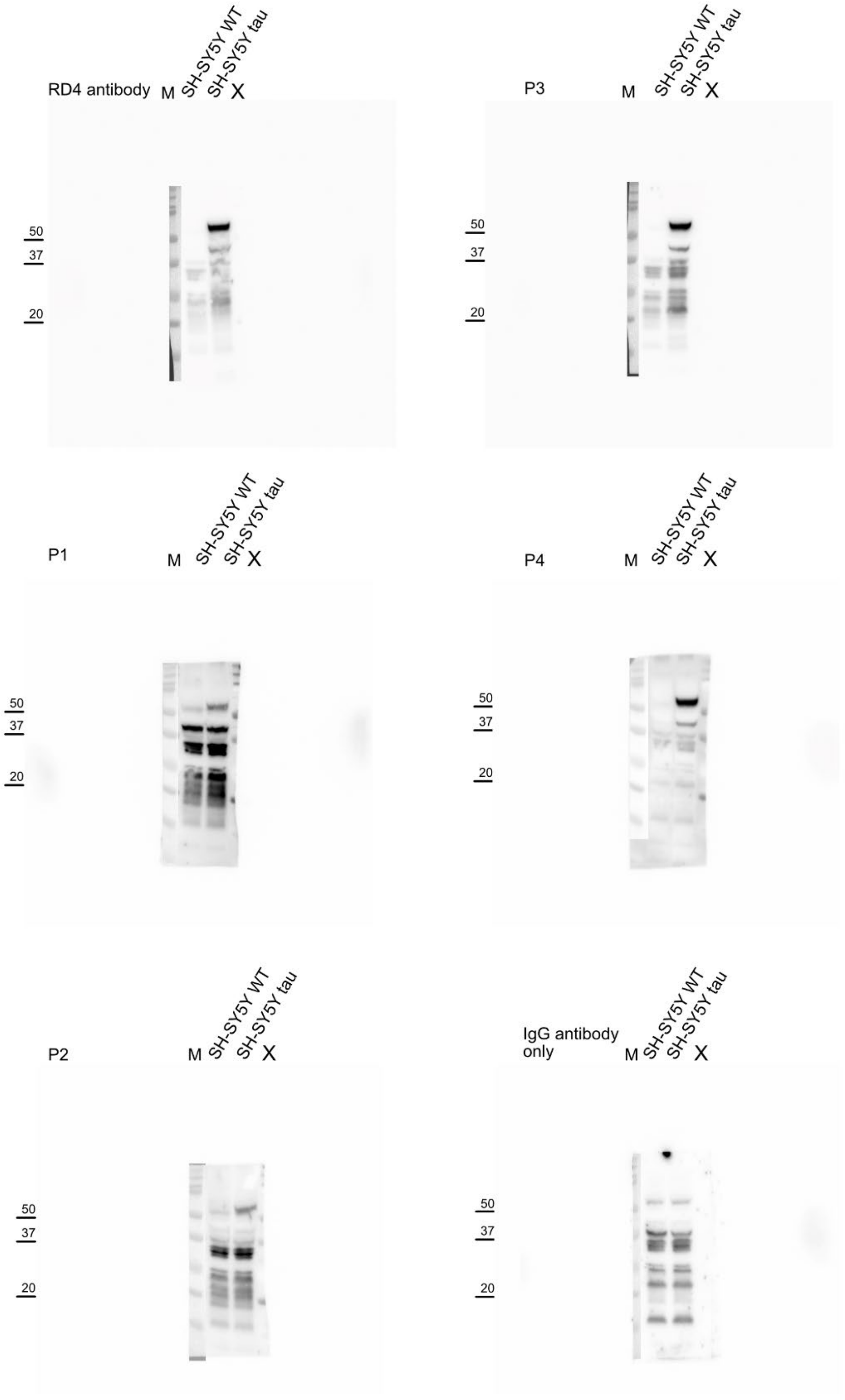

X not used in Figure

Fig. 2F: Raw WB images acquired by the Vilber Fusion Solo imaging system.

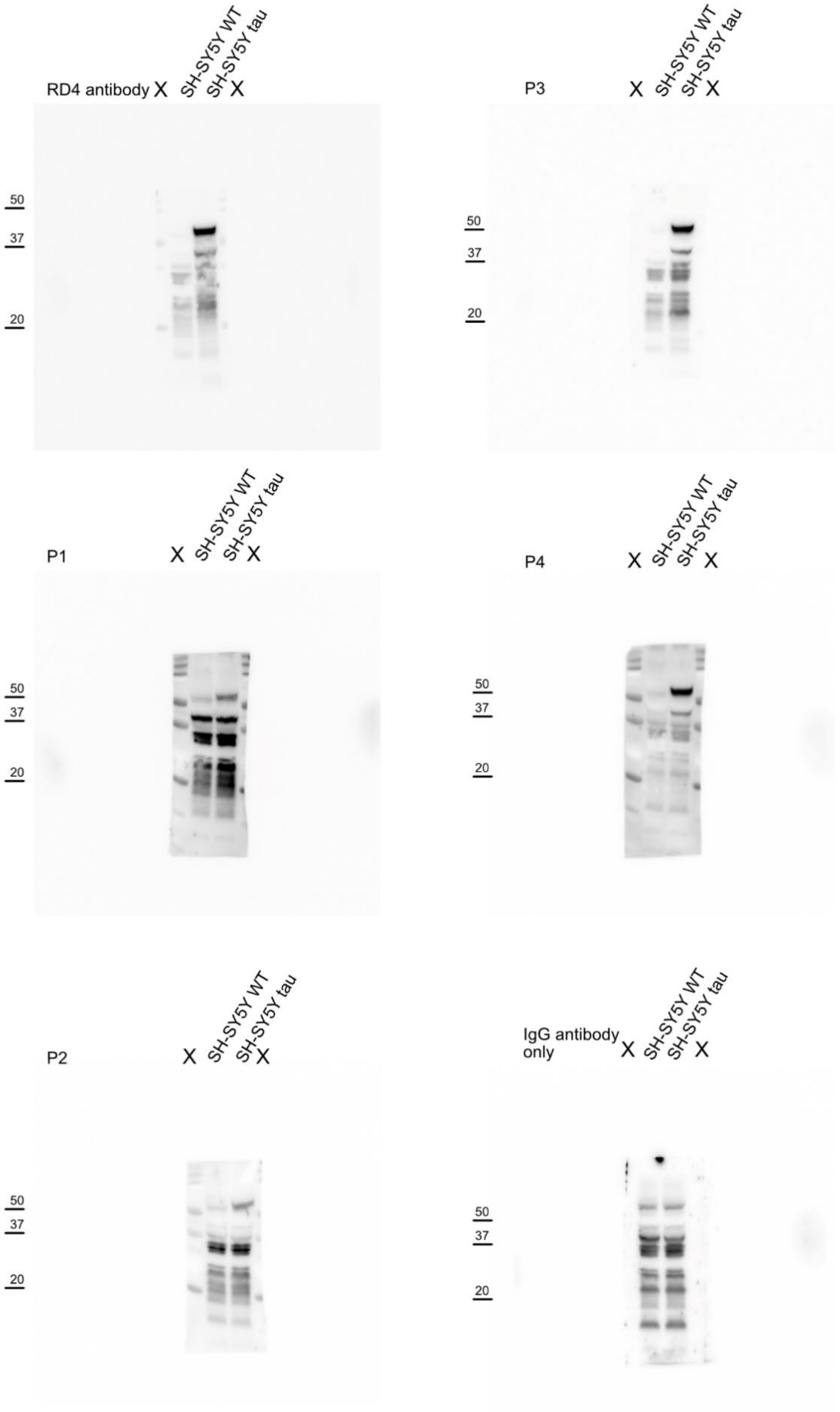

X not used in Figure
