# Supplementary figures and images for: Large-scale seroepidemiology uncovers nephro-urological pathologies in people with tau autoimmunity
Source: PLoS Biol. 2025 Nov 26;23(11):e3003488. doi: 10.1371/journal.pbio.3003488 (PMC12685212; doi:10.1371/journal.pbio.3003488)

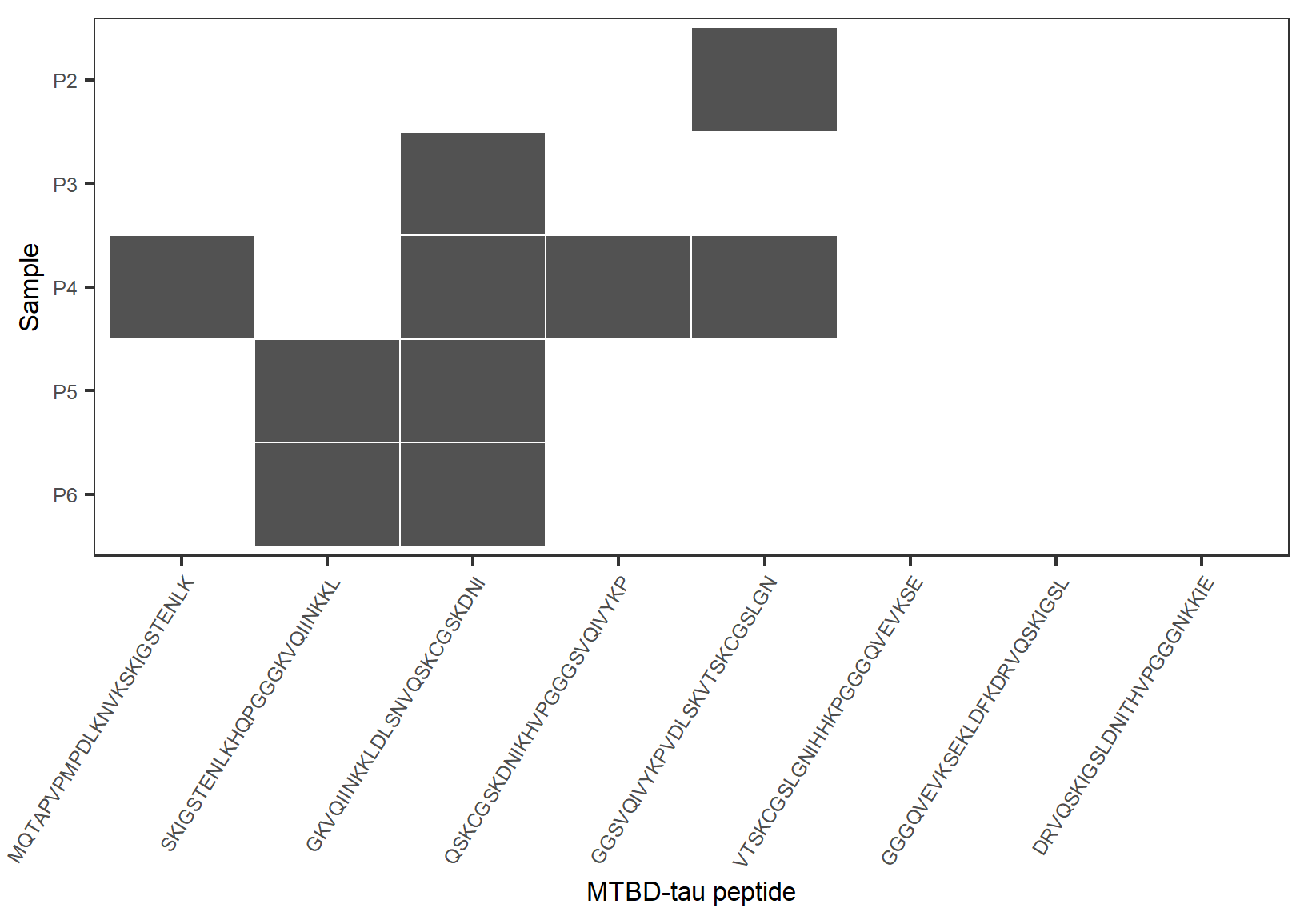

Supplement: S1 Fig — Epitope mapping of the same samples used in the assay shown in Fig 3C and 3D against eight 25mer MTBD-tau peptides overlapping 10 residues. (TIFF) [file pbio.3003488.s007.tiff]

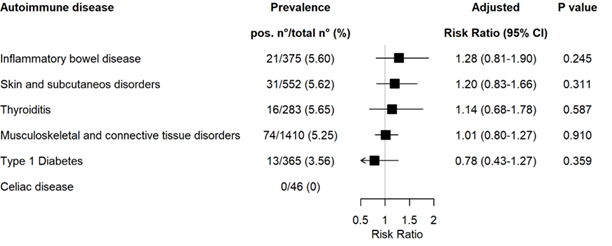

Supplement: S2 Fig — Forest plot showing the risk ratios and 95% CI (I bars) for the detection of tau autoantibodies in plasma samples of patients according to ICD-10 diagnosis of 6 different autoimmune diseases. aRR and 95% CI were estimated using log-binomial regression multivariate models including the respective variable, age, and sex. (TIFF) [file pbio.3003488.s008.tiff]
