# Supplementary material for: Large-scale seroepidemiology uncovers nephro-urological pathologies in people with tau autoimmunity
Source: PLoS Biol. 2025 Nov 26;23(11):e3003488. doi: 10.1371/journal.pbio.3003488 (PMC12685212; doi:10.1371/journal.pbio.3003488)
Supplement: S3 Fig — The alignment results from a BLASTP search with the BLOSUM62 substitution matrix (https://blast.ncbi.nlm.nih.gov/). The search was conducted against proteins present in the human kidney and urinary proteome (UniProt/Swiss-Prot). The alignment revealed 28% sequence identity. (PDF) [file pbio.3003488.s009.pdf]

```
MTBD-tau  13   VKSKIGSTENLKHQPGGGKVQIINKKLDLSNVQSKCGSKDNIKHVPGGGSVQIVYKPVDL  72
               +K K+ S EN+    GGG +Q + +  DL N   KC        V  G  + +   PV
NAMPT     368  MKQKMWSIENIAFGSGGGLLQKLTR--DLLNCSFKCSYV-----VTNGLGINVFKDPVAD  420

MTBD-tau  73   SKVTSKCGSLGNIHHKPGGGQVEVKSEKLDFKDRVQSKIGSL  114
                   SK G L ++H  P G   V ++  K D ++  Q  + ++
NAMPT     421  PNKRSKKGRL-SLHRTPAGNFVTLEEGKGDLEEYGQDLLHTV  461
```
