# Supplementary material for: Large-scale seroepidemiology uncovers nephro-urological pathologies in people with tau autoimmunity
Source: PLoS Biol. 2025 Nov 26;23(11):e3003488. doi: 10.1371/journal.pbio.3003488 (PMC12685212; doi:10.1371/journal.pbio.3003488)
Supplement: Attachment — Submitted filename: 157448_1_rebuttal_3470463_t0m8kg.docx [file pbio.3003488.s010.docx]

**Manuscript number:** RC-2025-03001

**Corresponding author(s):** Simone Hornemann, Adriano Aguzzi

## 1. General Statements [optional]

We thank the reviewers for their thorough and insightful evaluation of our manuscript, and for acknowledging its conceptual significance, methodological rigor, and relevance to both clinical practice and research. Below, we provide a point-by-point response to the reviewers’ comments. We have revised the manuscript accordingly.

**Reviewer #1**

**Evidence, reproducibility and clarity**

Summary: 
The authors present a large-scale seroepidemiological study analyzing 40,497 human plasma samples from hospital patients and healthy blood donors to detect the presence of IgG autoantibodies against the microtubule-binding domain of tau protein (MTBD-tau). Using a miniaturized, high-throughput ELISA platform, they identify a surprising prevalence of anti-tau autoantibodies (ατ+), particularly among older individuals, hospital patients, and women. Notably, they find no association with neurodegenerative diseases, but rather strong correlations with renal and urological disorders. These associations are supported by epitope specificity assays, tau aggregation inhibition assays, ICD-10 diagnosis analysis, and laboratory biomarkers.

Major Comments: 
Are the key conclusions convincing?

**Comment 1:** Yes. The main conclusions, particularly the extraneural association with renal and urinary disorders, are well supported by robust statistical analyses, extensive sampling, and biological validation.

***Response 1****: We appreciate the reviewer’s positive assessment of our core findings and the validation strategy employed. No changes were made in response to this point.*
 
**Comment 2:** Should the authors qualify any claims as speculative or preliminary? 
The authors generally interpret their findings conservatively and explicitly state that their design does not allow causal inference. However, the distinction between physiological (natural) and pathological autoantibodies could be more explicitly articulated in the Introduction and Discussion.

***Response 2****: We have added text to the Introduction and Discussion sections to highlight the distinction between physiological and pathological autoantibodies which can indeed depend on the health status of the individual.*

Would additional experiments be essential? 
**Comment 3:** While an independent replication cohort would strengthen the findings, it is not essential for publication of the current manuscript. The biochemical validation (e.g. specificity, lack of polyreactivity, functional inhibition of tau aggregation) is thorough.

***Response 3:*** *This point was already included in the Discussion. We have now revised the text to highlight it more clearly. We also thank the reviewer for recognizing the thoroughness of our validation.*

Are proposed experiments realistic (cost/time)? 
**Comment 4**: Future validation studies, e.g. in a prospective or longitudinal clinical setting, could realistically be conducted over 12 months with moderate resource requirements, depending on the infrastructure. Such studies are recommended, but not critical at this stage.

***Response 4:*** *We agree that future validation studies in a prospective and longitudinal design would be valuable. While such studies are important for future translational validation, we believe they are not critical to the current conclusions of our work, which are well supported by the present data.*

Are the data and methods presented in a reproducible way? 
**Comment 5*:*** Yes. The manuscript includes detailed descriptions of the ELISA setup, antigen preparation, antibody purification, and statistical models. Assays were internally replicated, and the microELISA platform was validated for reproducibility.

***Response 5:*** *We appreciate the reviewer’s positive assessment of our methodology.*

Are experiments adequately replicated and is statistical analysis appropriate? 
**Comment 6**: Yes. Key experiments include technical replicates, and the authors use appropriate statistical methods. Their approach to adjusting for age and sex confounders is methodologically common.

***Response 6:*** *We also thank the reviewer for recognizing the adequacy of our experiments and statistical approach.*
 
Minor Comments: 
**Comment 7:** The distinction between natural and disease-associated autoantibodies should be emphasized more clearly in the narrative to avoid misinterpretation.

***Response 7:*** *We acknowledge this and have further expanded on it in the Introduction and Discussion sections.*

**Comment 8:** The referencing is appropriate and up-to-date, covering both historical and recent literature on tau pathology and natural autoimmunity.

***Response 8****: We also thank the reviewer for acknowledging the appropriateness of the references used.*
 
**Comment 9:** The text is generally well written and logically structured. Minor editorial tightening could further improve clarity, especially in the methods section.

***Response 9:*** *We thank the reviewer for pointing this out. In response, we have streamlined the Methods section to enhance readability.*

**Significance**

This manuscript represents a conceptual advance, identifying a previously unrecognized association between tau autoimmunity and extraneural diseases, specifically renal and urinary tract disorders. It challenges the assumption that tau-related autoimmunity is solely or primarily relevant to neurodegeneration. Moreover, the hypothesis-free "reverse immunopathology" approach applied at population scale is innovative and broadly applicable to other autoantigens. 
 
Placement within existing literature: 
The findings contrast with earlier reports that focused on tau autoantibodies in Alzheimer's and Parkinson's disease patients (e.g., Bartos et al., 2018; Kronimus et al., 2016). They are aligned, however, with emerging evidence that tau is expressed in non-neuronal tissues including kidney and bladder (e.g., Rinschen et al., 2018), providing biological plausibility for the observed extraneural associations. 
 
Audience and impact: 
This work will be of interest to: 
- Immunologists studying natural autoantibodies and systemic immunity 
- Nephrologists and researchers of kidney disease 
- Neurodegeneration researchers and biomarker developers 
- Clinicians involved in tau immunotherapy trials (due to potential extraneural effects) 
 
Reviewer expertise: 
Fields of expertise: Neuroimmunology, autoantibodies, protein biochemistry, biomarker development, neurodegenerative diseases

**Reviewer #2**

**Evidence, reproducibility and clarity**

Natural autoantibodies are immunoglobulins that recognize self-antigens in the absence of external antigenic stimulation. While anti-tau autoantibodies have been detected in the plasma of patients with Alzheimer's disease (AD) and Parkinson's disease, they have also been observed in individuals without neurodegenerative conditions. To further explore this phenomenon, the present study employed a two-site cross-sectional design to analyze anti-tau autoantibodies in a large cohort of patients and controls. The authors report that anti-tau autoimmunity is highly specific and unexpectedly prevalent, with its frequency increasing with age. Notably, the study identifies a previously unrecognized association between natural anti-tau autoantibodies and a syndrome characterized by renal and urinary tract disorders. This novel link between anti-MTBD-tau antibodies and non-neurological pathologies may have important implications for both researchers and clinicians.

However, several concerns should be addressed: 
 
**Comment 1.** Clarify the term "MTBD-tau": presumably referring to peptides derived from the microtubule-binding domain of tau. This should be clearly defined in the manuscript.

***Response 1:*** *We have clarified the definition of the term “MTBD-tau” in the beginning of the Results section (PAGE 6, lines 81-82) detailing that it corresponds to the microtubule binding domain of tau (residues 244–372 relative to 2N4R human tau).*

**Comment 2.** Provide epidemiological context: It would be informative for readers if the authors could report approximate population-level frequencies of natural autoantibodies against other intracellular proteins, to contextualize the prevalence of anti-tau autoantibodies.

***Response 2:*** *To provide epidemiological context, we now included seroprevalence estimates of natural autoantibodies against selected neuronal intracellular antigens as reported by Dahm L. et al, 2014. Specifically, seroprevalences of autoantibodies varied between 0.4% (anti-Ma1) and 2.0% (anti-amphiphysin). These data were added to the Discussion. We believe this additional context strengthens the manuscript by situating our findings within the broader neuronal immunological landscape.*

**Comment 3.** Address the plausibility of anti-MTBD-tau antibodies in circulation: The microtubule-binding domain of tau is typically buried in the native conformation and is not expected to be exposed under physiological conditions. Moreover, extracellular tau in plasma and cerebrospinal fluid (CSF) is primarily composed of N-terminal fragments, as the protein is often cleaved in the repeat domain region. The detection of antibodies against the MTBD region raises the possibility of cross-reactivity with other proteins that may share structural or sequence similarities.

***Response 3:*** *We agree that under physiological conditions, MTBD-tau is typically sequestered in the intracellular environment and largely inaccessible. Moreover, a newly identified N-terminal tau fragment measured in plasma has a high specificity for AD populations.*

*Despite this, in our current study, we consistently detected natural autoantibodies against the MTBD region of tau in a substantial subset of individuals. To rigorously assess the specificity of these antibodies and rule out cross-reactivity, we performed a series of orthogonal validation experiments, including testing for cross-reactivity using microELISA in over 10’000 patients against two other neuronal antigens (Figure 1F and 1G), competition assays with recombinant tau fragments and other unrelated proteins (Figure 2C), polyreactivity testing against five other unrelated antigens (Figure 2D), immunofluorescence stainings (Figure 2E) and western blotting (Figure 2F). These experiments did not reveal significant cross-reactivity with other known autoantigens (please see Methods and Results section). Thus, we believe the signal reflects specific recognition of MTBD-tau epitopes.*

*We acknowledge in the Dicussion that the presence of these antibodies in circulation may appear counterintuitive from a structural standpoint. However, one possible explanation is that MTBD-tau fragments may be transiently exposed or released in the context of non-neurological cellular turnover or tissue damage, particularly in peripheral tissues. This idea is supported by our observed association between anti-MTBD-tau antibodies and renal/urological pathology, but not with neurological disorders (Figures 5 and 6) suggesting that tau may be encountered by the immune system in non-neuronal contexts.*

**Comment 4.** Evaluate potential cross-reactivity with kidney-related antigens: Given the unexpected association between anti-tau autoimmunity and renal/urinary symptoms, it is critical to explore whether these antibodies might bind to endogenous antigens in the kidney or urinary tract. Cross-reactivity studies should be considered to support or refute this hypothesis.

***Response 4:*** *We appreciate the reviewer’s insightful comment regarding the potential for cross-reactivity between anti-MTBD-tau antibodies and endogenous renal and/or urological antigens. We fully agree that this is an important consideration, especially in light of the novel association observed between anti-tau autoimmunity and kidney/urinary disorders.*

*To address the concern of non-specific binding or cross-reactivity, we conducted extensive orthogonal testing as mentioned above. These experiments did not reveal cross-reactivity with other known neuronal or systemic autoantigens, supporting the antigenic specificity of the anti-**MTBD-tau antibodies detected in our cohort.*

*However, we acknowledge that our current study did not directly assess reactivity against kidney- or renal/urinary tract-specific proteins. In response to the reviewer’s suggestion, we performed an in silico analysis comparing the MTBD-tau sequence to proteins expressed in the human kidney and urinary proteome (UniProt/Swiss-Prot) using BLASTP. The protein with the highest sequence identity (~28%) was nicotinamide phosphoribosyltransferase (NAMPT), a metabolic enzyme with widespread tissue expression. Nevertheless, such sequence-based analysis is limited to detect linear or conformational epitopes that might mediate cross-reactivity. The sequence alignment has been included as Supplementary Figure 4, and corresponding text has been added to the Discussion.*

**Comment 5.** Additionally, the authors address the issue that in renal disease, there is a strong possibility of reverse causation, as urinary protein exposure to the immune system and decreased clearance of autoantibodies can be contributing factors. 

***Response 5:*** *Indeed, it is well recognized that in renal disease, compromised glomerular filtration and altered immune function can lead to increased systemic exposure to intracellular antigens due to tubular damage and urinary protein leakage, and reduced clearance of circulating immunoglobulins, including natural autoantibodies. These mechanisms may contribute to increased detection of anti-tau antibodies in individuals with renal impairment, independent of any primary immunologic trigger.*

*We have revised the Discussion to acknowledge the possibility of reverse causality. While our cross-sectional design precludes determination of causality or directionality, we note that:*

- *Anti-MTBD-tau autoantibodies were observed even in individuals without overt renal disease, though prevalence increased with age and renal comorbidity.*
- *There was no enrichment of common autoimmune disorders (Supplemental Figure 3), suggesting that general immunoglobulin retention is unlikely to fully account for our findings.*

*Nonetheless, we agree that prospective studies with longitudinal antibody profiling and kidney function monitoring will be essential to clarify whether the renal association reflects secondary immune activation, reduced clearance, shared antigen exposure, or a distinct immunological phenomenon.*

**Comment 6:** Despite multivariate analysis, the persistence of multiple significant differences between disease and antibody positivity even after Bonferroni correction raises concerns about Type I errors stemming from multiple comparisons.

***Response 6:*** *We agree that large-scale association testing carries an inherent risk of false positives due to multiple comparisons. To mitigate this, we applied a stringent Bonferroni correction and conducted multivariate regression models adjusting for relevant covariates (age, sex). Importantly, several associations, particularly between anti-MTBD-tau antibodies and renal/urological phenotypes, remained statistically significant even under this conservative correction and using different statistical approaches (ICD-10 diagnosis, laboratory parameters), supporting the robustness of these findings. We now emphasize in the Discussion that while our associations are statistically strong, they require replication in independent cohorts to confirm biological/clinical significance.*

**Comment 7**: The primary outcome, defined by a cutoff of −log10(EC50) {greater than or equal to} 1.8, lacks clear biological meaning.

***Response 7:*** *We agree with the referee that the threshold used to define antibody positivity may not be entirely intuitive. The cutoff was derived empirically from assay validation experiments (Emmenegger M, et al, iScience. 2023;26(2):105928 and Figure 1E)) and reflects the optimal compromise between sensitivity and specificity for our hypothesis-generating primary assay. This cutoff corresponds approximately to an EC_50_ ≥ 2^6^, or nominal dilution of > 1/64 (see Results section) representing the inflection point above background and below saturation in our serial dilution assays. We have now more clearly described this in the Results section.*

**Significance**

The study identifies a previously unrecognized association between natural anti-tau autoantibodies and a syndrome characterized by renal and urinary tract disorders. This novel link between anti-MTBD-tau antibodies and non-neurological pathologies may have important implications for both researchers and clinicians.

**Reviewer #3**

**Evidence, reproducibility and clarity:** Naturally-occurring antibodies to various neuronal proteins have been previously reported, generating considerable debate about their pathological or protective significance. In this manuscript, Hornemann, Aguzzi and colleagues report a hospital-based study of the prevalence of naturally occurring antibodies to the microtubule-binding region (MTBR) of tau protein in a large set of over 40,000 clinical biochemistry plasma samples from hospital patients and healthy blood doners. Excluding non-informative samples, data from over 30,000 samples was collected and analysed and naturally occurring tau antibodies (to the MTBR) were found in approximately 5% of hospital patients.

The tau protein is centrally involved in the pathogenesis of several neurodegenerative diseases, including Alzheimer's disease and frontotemporal dementia, and formation of intracellular aggregates of tau is a key pathology in those diseases. As is commented on by the authors, a number of tau antibodies have been developed as candidate treatments for tauopathies such as Alzheimer's, with limited evidence for therapeutic efficacy in human clinical trials to date - although it must be emphasized that the antibodies that previously failed in trials were directed to the N-terminal region of tau.

Major comments:

**Comment 1:** The paper is framed as being relevant to the development of therapeutic antibodies to tau for the treatment of Alzheimer's disease and other tauopathies. However, it is not clear from the titres reported here how those concentrations compare to the concentrations in plasma of therapeutics antibodies delivered iv or subcutaneously. Given that the typical concentration of antibodies in the CNS is less than 0.1% of that in plasma, this information would be helpful for interpreting the relevance of the findings presented here for the therapeutic potential of passive immunity from exogenous antibodies.

***Response 1:*** *We thank the reviewer for raising this important point regarding the translational relevance of our findings to tau-targeting immunotherapy. While our study identifies the presence of naturally occurring anti-MTBD-tau antibodies in the general population and association with kidney and urinary disorders, we emphasize that these autoantibodies were not associated with a reduced risk of neurodegenerative diseases in our cohort. As such, we cannot conclude that antibodies against MTBD-tau confer a protective effect against tauopathies such as Alzheimer’s disease. On the other hand, the association with kidney and urinary disorders could potentially raise a concern, that tau autoantibodies could drive extra-neural pathology. However, we would like to emphasize this is just a hypothesis raised by this study and that further studies, namely longitudinal studies, would be required to assess causality (this limitation of our study has been highlighted in the Discussion section).*

**Comment 2:** The longest CNS isoform of tau is 441 amino acids, but the study only analysed the MTBR, which is less than 1/3 of the protein. As such, while the qualitative findings around the frequency of anti-MTBR tau antibodies in the population are of interest, it is not an accurate measure of the true frequency of anti-tau antibodies in the population. Have the authors carried out studies using full-length tau in a smaller set of samples?

***Response 2:*** *Our primary analyses focused on MTBD-tau. Our choice to concentrate on this region of tau was guided by its central role in tau aggregation and its pathological relevance in tauopathies. We agree that this approach does not capture the full spectrum of potential anti-tau immune responses, particularly against the N-terminal and C-terminal regions and also of post-translationally modified tau. This limitation is noted in the Discussion section.*

*To address the reviewer’s question directly: we also investigated plasma anti-full-length-tau autoantibodies in a subset of samples (47 patients with AD and 68 non-AD controls). Also in this cohort, we didn’t see any significant difference in reactivity between the plasma samples from AD patients and non-AD controls (Figure 5B).*

**Comment 3:** Related to this, tau is expressed in both the CNS and PNS, and fragments of tau from both regions of the nervous system are found in plasma, with PNS-derived tau much more abundant. It would be of interest to compare the prevalence of naturally occurring antibodies to PNS-derived tau (big-tau) with the findings reported here, which relate to all forms of tau.

***Response 3:*** *We thank the reviewer for this insightful and biologically relevant comment. Indeed, big tau, the high–molecular weight isoform expressed primarily in the PNS, differs structurally from CNS tau in that it includes an additional large exon (exon 4a). Our current study focused on antibodies targeting the microtubule-binding domain of tau, which is conserved across all tau isoforms, including both CNS tau and big tau. As such, our assay does not distinguish between antibodies generated in response to CNS-derived versus PNS-derived tau antigens. We agree that this distinction would be interesting, particularly given the possibility that circulating tau, especially big-tau, may serve as a primary immunogen for natural antibody production. Such serological studies using recombinant big tau, including the exon 4a region, could indeed be of interest in the future. We have added this point to the revised Discussion.*

**Comment 4:** For context, the authors should discuss in more detail how common natural autoantibodies to neuronal proteins are, within the general population. Is tau unusual in this respect? From the discussion it appears to be so, but this is not thoroughly dealt with.

***Response 4:*** *As mentioned above in response to reviewer #2 (comment 2), we have revised the Discussion section to include seroprevalence estimates of natural autoantibodies against selected neuronal intracellular antigens.*

**Comment 5:** Samples were designated as having a high titre of anti-MTBR tau based on an empirical threshold. How was this threshold determined?

***Response 5:*** *As discussed in our response to reviewer #2, comment 7, the threshold for defining antibody positivity was derived empirically from assay validation experiments (Figure 1E).*

**Comment 6:** Purified anti-tau antibodies had EC50 values that are several orders of magnitude lower than that of a monoclonal developed against MTBR-tau. Does this suggest they are low affinity antibodies? If so, what is the significance of this?

***Response 6:*** *We thank the reviewer for this insightful question. The purified anti-MTBD-tau autoantibodies from human plasma exhibited higher EC_50_ values (i.e., lower apparent binding affinity) compared to a commercial high-affinity monoclonal antibody (RD4) developed against the same domain. This is consistent with the literature-discussed characteristic of natural autoantibodies, which are typically low-affinity, polyreactive, and largely germline-encoded. The relatively low affinity observed here likely reflects the fact that these autoantibodies arise without overt antigen-driven affinity maturation (supported by the evidence that most tau autoimmune responses were polyclonal (please see Figure 2H and 2I) and may instead represent part of the natural antibody repertoire maintained by B-1 cells or marginal zone B cells. Their functional role is thought to center on immune surveillance, debris clearance, and homeostatic regulation, rather than high-specificity immune defense. In this context, the low affinity of the anti-MTBD-tau antibodies supports the interpretation that they are likely homeostatic and unlikely pathological. We have clarified this point in the revised Discussion and emphasize that these autoantibodies can also reflect physiological autoimmunity with low affinity and no clear link pathology.*

**Comment 7:** The inclusion of data demonstrating that these antibodies can inhibit tau aggregation are interesting, but unsurprising, as any antibody to tau's MTBR will have this property. It's not clear what the authors' point is here, as no conclusion is drawn from this finding.

***Response 7:*** *We agree that antibodies targeting the microtubule-binding domain of tau would generally be expected to interfere with aggregation, as this domain is critical for aggregation nucleation and elongation. Our primary motivation for including the in vitro tau aggregation assay was not to demonstrate a novel function, but rather to confirm that the natural antibodies we detected are able to bind to tau and are functionally competent in modulating in vitro aggregation assay. Given the novelty approach of our study, we used this assay (and others) to extensively demonstrate that the detected anti-tau autoantibodies are able to specifically bind to MTBD-tau. We have added a reference to the specific inhibition of tau aggregation in the concluding remarks about this assay in order to clarify the goal and interpretation of the assay (Results section).*

***Comment 8:*** The data reported in Figure 5 exploring differences in the presence of MTBR tau antibodies in AD patients and controls are problematic in their reporting and interpretation. Extrapolating the 5% prevalence figure for tau antibodies in hospital patients, 2-3 of the 47 AD patients would be predicted to have antibodies vs 3-4 of the 68 controls, using the criteria reported in this paper. If tau antibodies are protective, one would need far larger patient numbers to detect any differences between these populations - the authors should include calculations of the power of this experiment, and what sort of differences would need to be present for them to the detectable using these numbers of samples. Instead the authors have reported reactivity as a continuous variable across each cohort. Why was this done, rather then the binary classification used for the presence and absence of tau antibodies?

***Response 8:*** *We appreciate this thoughtful and important comment raising valid concerns about the comparison of tau autoantibody reactivity in AD and controls. We fully agree that the sample sizes used in this comparison (47 AD patients and 68 age-matched controls) are not sufficient to robustly test for small-to-moderate differences in anti-MTBD-tau antibody prevalence between groups. As rightly mentioned, extrapolating from the estimated 5% overall frequency, the expected number of tau-seropositive individuals in each group would be very small, and detecting statistically significant group-level differences under such conditions would require substantially larger cohorts.*

*A power analysis indicates that, with our current sample size, the study has a power of 80% to detect a large difference in prevalence (e.g., ≥25% vs. 5%), but only about ~15% power to detect small differences (e.g., 10% vs. 5%). Thus, the absence of a statistically significant difference in our data should not be interpreted as definitive evidence of no association. The reason we included such a low number of participants was because this was a convenience sample of the plasma available in our biobank and no more samples were available to us at the time. We agree with the reviewer that this is a major limitation and have added this in the Results section and in the Discussion section.*

*Regarding the use of a continuous metric (–log_10_ [ EC_50_]), we chose this approach because:*

- *we tested the plasma samples not only for IgG autoantibodies against MTBD-tau (as in the primary screen) but also for IgG autoantibodies against full-length-tau and IgA autoantibodies for which a threshold for positive reactivity we did not establish in a large-scale primary screen*
- *it allowed us to examine the full distribution of reactivity in both groups without applying an arbitrary threshold we could not validate in a large-primary screen*
- *it would provide greater sensitivity for detecting sub-threshold differences that might be biologically relevant but would be missed in binary classification.*

**Comment 9:** While of interest, the correlation between tau antibodies and kidney disease raises many questions, not least the breadth of renal conditions that seem to be involved, ranging from infection to chronic renal failure. Given this, it is challenging to see how tau antibodies could be causal for all of these conditions, rather than a consequence of various forms of renal damage. This point would benefit from more in depth discussion in the manuscript. While beyond the scope of this paper, one potential next step would be a study of tau antibodies in renal failure patients, for example.

***Response 9:*** *We agree that the diversity of renal and urinary conditions associated with anti-MTBD tau antibodies, including infections/inflammation and chronic kidney disease, makes it unlikely that these antibodies are directly pathogenic across all categories. Rather, our findings are more plausibly explained by secondary mechanisms, including exposure of sequestered intracellular proteins, such as tau, due to renal tissue injury or inflammation, increased immune access to urinary proteins due to epithelial barrier disruption, or reduced clearance of circulating antibodies in the context of renal dysfunction. We have now expanded the Discussion (page 13 and 14) to acknowledge these possible reverse causation and/or epiphenomenal associations as possible contributors for this association. We also agree that further mechanistic investigation is warranted (also mentioned in the Discussion), but clearly beyond the scope of this study.*

**Comment 10:** A potentially important finding is the interference of these antibodies with detection of plasma tau, which is becoming widely adopted as a biomarker for Alzheimer's disease diagnosis and staging. However, the way this experiment was performed does not reflect the nature of endogenous plasma tau, which is largely composed of N and C/MTBR region fragments. So while it is possible that the MTBR antibodies could interfere with biomarker assays, it is not as straightforward as modelled here using full length tau protein.

***Response 10****: We agree that nowadays the tau species that appear to provide a better specificity for the diagnosis and monitoring of AD corresponding to specific posttranslational modifications and isoforms. Therefore, our assay using MTBD-tau autoantibodies and recombinant full-length tau likely does not fully capture the complexity or heterogeneity of tau fragments present in the plasma of AD patients. Our intention with this experiment was to model a proof-of-principle scenario to explore whether natural anti-MTBR antibodies have the potential to interfere with immunoassays, especially those employing capture or detection antibodies that target epitopes within or near the MTBD region. While this setup is a simplification, we agree with the reviewer that the results should not be over-interpreted, and we have clarified this point in the revised Discussion (page 14) mentioning that assay interference is epitope- and diagnostic assay-specific and would depend on whether MTBR epitopes are actually present and accessible in the analyte forms targeted by specific diagnostic assays.*

**Significance:**

Overall, the paper reports an interesting cross-sectional study that is technically thorough and well reported. However, the framing of the paper is confusing: the authors briefly mention in the introduction that tau antibodies have previously been detected in healthy individuals, as well as people with Alzheimer's and Parkinson's disease, and raise the question of the possible relationship between such antibodies and risk of developing neurodegenerative disease. The study reported here does not appear to have been designed appropriately to answer this question, instead finding a positive correlation between tau antibody positivity and the renal/urinary diseases. This is an interesting finding, but, as the authors point out, cannot establish causality.

In summary, this is an interesting dataset on the prevalence of autoantibodies to a specific domain of the tau protein, but which is somewhat confused in its presentation, data interpretation and main conclusions.
